# Bloodstream infection with *Acinetobacter baumanii* in a *Plasmodium falciparum* positive infant: a case report 

**DOI:** 10.1186/s13256-020-02648-7

**Published:** 2021-02-05

**Authors:** Charity Wiafe Akenten, Kennedy Gyau Boahen, Kwadwo Sarfo Marfo, Nimako Sarpong, Denise Dekker, Nicole Sunaina Struck, Lawrence Osei-Tutu, Juergen May, John Humphrey Amuasi, Daniel Eibach

**Affiliations:** 1 Kumasi Centre for Collaborative Research in Tropical Medicine, P.O Box PMB KNUST, Kumasi, Ghana; 2 Bernhard Nocht Institute for Tropical Medicine, Hamburg, Germany; 3 Agogo Presbyterian Hospital, Asante Akyem, Ghana; 4 School of Public Health, Kwame Nkrumah University of Science and Technology, Kumasi, Ghana

**Keywords:** Malaria, Coinfections, *Acinetobacter baumanii*, Bacteremea, Case report, Ghana

## Abstract

**Background:**

The increasing incidence of multi-antibiotic-resistant bacterial infections, coupled with the risk of co-infections in malaria-endemic regions, complicates accurate diagnosis and prolongs hospitalization, thereby increasing the total cost of illness. Further, there are challenges in making the correct choice of antibiotic treatment and duration, precipitated by a lack of access to microbial culture facilities in many hospitals in Ghana. The aim of this case report is to highlight the need for blood cultures or alternative rapid tests to be performed routinely in malaria patients, to diagnose co-infections with bacteria, especially when symptoms persist after antimalarial treatment.

**Case presentation:**

A 6-month old black female child presented to the Agogo Presbyterian Hospital with fever, diarrhea, and a 3-day history of cough. A rapid diagnostic test for malaria and Malaria microscopy was positive for* P. falciparum* with a parasitemia of 224 parasites/μl. The patient was treated with Intravenous Artesunate, parental antibiotics (cefuroxime and gentamicin) and oral dispersible zinc tablets in addition to intravenous fluids. Blood culture yielded* Acinetobacter baumanii*, which was resistant to all of the third-generation antibiotics included in the susceptibility test conducted, but sensitive to ciprofloxacin and gentamicin. After augmenting treatment with intravenous ciprofloxacin, all symptoms resolved.

**Conclusion:**

Even though this study cannot confirm whether the bacterial infection was nosocomial or otherwise, the case highlights the necessity to test malaria patients for possible co-infections, especially when fever persists after parasites have been cleared from the bloodstream. Bacterial blood cultures and antimicrobial susceptibility testing should be routinely performed to guide treatment options for febril illnesses in Ghana in order to reduce inappropriate use of broad-spectrum antibiotics and limit the development of antimicrobial resistance.

## Introduction

Bloodstream infections (BSI) are major causes of hospitalization and mortality worldwide [1, 2]. Globally, neonatal sepsis causes an estimated 750,000 deaths annually with the highest mortality in Sub-Saharan Africa (SSA) [3]. Among pediatric populations in SSA, annually, an estimated 5.29–8.73 million disability-adjusted life years are lost with a yearly economic burden between 10 and 469 billion US-dollars [3]. Therefore, accurate identification of the etiological agents and the determination of antibiotic resistance patterns is essential for informing treatment, surveillance and preventive measures [2].

Overlapping clinical symptoms of febrile illnesses make it difficult for medical doctors to diagnose bacterial infections. Furthermore, the increasing burden of bacterial multi-resistance both limits the choice of antibiotics and makes empirical use of broad-spectrum antibiotics [4]. Blood cultures are not routinely performed in health facilities in Ghana largely on account of economic constrains. Even when performed, the results come days later, when patients would have already received significant antibiotic treatment. Not knowing if or which bacterial pathogen is causing the infection results in the use of broad-spectrum antibiotics, which in turn promotes antibiotic resistance.

The World Health Organization (WHO) therefore recommends the combination of antimalarial and broad-spectrum antibiotics in children with severe malaria [5]. With rising numbers of circulating microbial pathogens, this recommendation may result in treatment failures in clinical practice due to microbial resistance to the antibiotic. This report presents a case of malaria with a bloodstream co-infection caused by *Acinetobacter baumanii*, where empirical treatment according to WHO recommendation [5] was unsuccessful, demonstrating a complex clinical challenge in rural SSA with limited diagnostic facilities.

## Case presentation

A previously well 6-month-old black female infant weighing 6.5kg, with a history of fever in the past, and with no known underlying conditions were referred to Agogo Presbyterian Hospital (APH) in the Asante Akim North District of the Ashanti region after having presented to a peripheral facility with 3 days of fever, diarrhea, and cough as reported by her mother. The diarrhea was watery and mucoid but non-bloody and was associated with episodes of non-projectile cough and vomiting. The infant was treated with homemade oral herbal preparations without improvement.

Upon presentation at the first point of call health facility, the child was managed with parenteral antibiotics, including intravenous cefuroxime, gentamicin and oral dispersible zinc tablets in addition to IV fluids according to the referral note. The child was subsequently referred to APH on account of her deteriorating health condition. Per the referral note, we were unable to ascertain if any laboratory tests or antimalarial treatment was initiated.

On examination, the child appeared ill, but was not febrile, with an axillary temperature of 36°C, pale, not jaundiced and adequately hydrated. Further examination focusing on the central nervous system found the child to be lethargic, prostrated (loss of neck control since the onset of illness) and with a Blantyre Coma Score (BCS) of 4/5 (Eye-opening E score 1/1; Motor score 1/2, and Vocal score 2/2). The patient was tachycardic with a heart rate of 146 beats per minute (bpm). The child was in severe respiratory distress with tachypnoea at rest (respiratory rate: 46 cycles per minute), grunting, nasal flaring and severe intercostal recession. Chest auscultation revealed adequate air entry with bilateral bronchial breath sounds. A rapid diagnostic test for malaria (Care Start Malaria, ACCESS BIO, Somerset, USA) was positive. The full blood count revealed moderate anemia with a hemoglobin concentration of 8.3 g/dL, leukocytosis (24.500/μL; neutrophils: 73.2%) and thrombocytosis (485,000/μL). Malaria microscopy was positive for *P. falciparum* with a parasitemia of 224 parasites/µL. Based on these findings the admission diagnosis was severe malaria with prostration and severe bronchopneumonia.

The patient was started on parenteral cefotaxime at 300 mg four times daily and 30 mg of gentamicin. Later on, the same day of admission, IV clindamycin at 40 mg four times daily for 4 days was administered. IV Artesunate was also started at 20 mg daily at time 0, at 12 h and 24 h per standard recommended treatment in addition to supportive care, being paracetamol when needed for pyrexia and supplemental oxygen for the respiratory distress. The patient was judged to be at a high risk of aspiration and was kept nil-by-mouth and maintained on IV fluids.

By day 2 of admission, the BCS had dropped to 2/5 (M-1, V-1, E-0). Her chest findings had not changed significantly. The same day, a diagnosis of cerebral malaria was included in her records. Treatment was continued and urine output monitored. The patient was started on a minimal enteral feed of 5 mL every 2 h. After 12 h the clinical state improved, with a decreased respiratory rate and Heart rate of 38 cpm and 126 bpm respectively, no signs of respiratory distress and an increasing BCS of 3/5 (E-1, M-1, V-1), which improved further three days into her admission to 5/5 (E-2, M-2, V-1). Her urine output was found to be less than 100 mL over the 24 h but improved following furosemide challenge.

## Follow-up and outcome

On day five following admission, the child continued to experience bouts of fever despite having completed antimalaria treatment and being on broad-spectrum antibiotics. All antibiotics were stopped for 48 h. On day seven, blood was taken for culture, full blood count and further malaria microscopy. The hemoglobin had dropped to 6.1 g/dL from initial 8.3 g/dL on admission, with persistent leukocytosis (24.500/μL) and thrombocytosis (295.000/μL). Malaria microscopy did not reveal any parasites in the thick and thin smear. IV ceftriaxone 500 mg daily for 72 h was administered while awaiting the blood culture results. She was also hemotransfused with 120ml of whole blood over 4 h at 30 mL per hour.

The blood culture sample was transported in a pediatric aerobic blood culture bottle (Beckton Dickinson, town, country) to the Kumasi Center for Collaborative Research in Tropical Medicine (KCCR) for analysis. The blood culture was performed as described by Hogan *et al.* [6]. The blood culture was positive after 24 h incubation and Gram staining showed gram-negative rods. Antimicrobial susceptibility testing was carried out using the Kirby-Bauer disk diffusion method with ampicillin (10 μg), ampicillin-sulbactam (20 μg), chloramphenicol (30 μg), co-trimoxazole (25 μg), ciprofloxacin (5 μg), ceftazidime (30 μg), gentamicin (10 μg), cefuroxime (30 μg) tetracycline (30 μg) following the European Committee on Antimicrobial Susceptibility Testing guidelines. The bacterial isolate was resistant to all antibiotics tested except for ciprofloxacin, gentamicin, and ampicillin-sulbactam (Table 1). The isolate was identified as a gram-negative rod and was later confirmed as *Acinetobacter baumannii* by Vitek MS (bioMerièux, Marseille, France) with a reliability of 99.9%**.**

**Table 1:** Result of the susceptibility pattern of the isolate

Antibiotic (disc concentration)	*Acinetobacter baumannii*
Ampicillin (10 μg)	R
Ampicillin-sulbactam (20 μg)	S
Chloramphenicol (30 μg)	R
Co-trimoxazole (25 μg)	R
Ciprofloxacin (5 μg)	S
Ceftazidime (30 μg)	R
Gentamicin (10 μg)	S
Cefuroxime (30 μg)	R
Tetracycline (30 μg)	R
Cefepime (30 μg)	R
Meropenem (10 μg)	R

*S* sensitive, *R* resistant, *I* intermediate

**Fig. 1.**
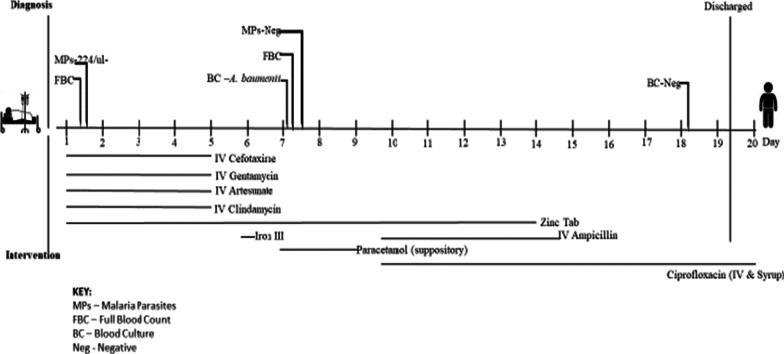
Clinical course of patient treatment

The blood culture result was communicated to the treating physician on the 9^th^ day after admission. Based on these results treatment was switched to a combination of parenteral ciprofloxacin (70 mg three times daily) for 72 h and gentamicin (250 mg 8 hourly for 4 days) which is readily available and affordable in addition to 250 mg of ampicillin which was later withdrawn. A second blood culture taken after 7 days of treatment was negative and the child had a consistently normal temperature. She was discharged after a total of 19 days of hospitalization on oral ciprofloxacin, which she was to continue for 5 days after discharge. The course of treatment of the patients has been summarized in Fig. 1.

## Discussion

This case reports the existence of concomitant malaria and *Acinetobacter baumannii* bacteremia in a 6-month old child. The child presenting to APH with a persistent fever after treatment and clearing of parasite, still harbored *Acinetobacter baumannii.* Malaria/BSI coinfections are suggested to be a major burden in SSA with the current rise of antibiotic-resistant bacteria [6, 7]. It has been demonstrated that bacterial infections significantly increase the risk of dying when co-diagnosed with severe malaria [8].

A study from Ghana showed that 5% of hospitalized children with a *Plasmodium* infection reveal a concomitant invasive BSI [6]. Similarly, systematic reviews found 6.5% of children with fever in SSA to be simultaneously positive for *Plasmodium* spp. and bacterial pathogens in blood cultures [7, 9]. A study in Kenya demonstrated that malaria infections predispose children in a region of high malaria endemicity to BSI [7, 10].

Malaria and bacteremia often present with similar clinical symptoms making differential diagnosis difficult [7, 8, 11, 12]. Therefore, it is essential to pay close attention to the identification of bacteremia in children with malaria by blood culture and antibiotic susceptibility testing.

This case report highlights the necessity of blood culture or alternative effective diagnostics for rural hospital settings in Ghana, that otherwise pose a significant health threat for pediatric patients. It also indicates that the WHO recommendation of broad-spectrum antibiotics and antimalarial treatment [5] may risk becoming increasingly unsuccessful due to bacteria being resistant to several commonly used antibiotics. Differential diagnosis of malaria and other febrile illness, such as BSI, therefore needs to be improved, especially in rural settings in SSA. Laboratory capacity in rural hospitals must be improved to aid in-patient diagnoses, particularly with regard to malaria co-infections. It wound be beneficial for blood cultures to be routinely performed by trained personnel for all children with persistent fever, even if tested positive for malaria [6].

The source of the isolated *Acinetobacter baumannii* strain in this 6-month-old child, hospital or community-acquired, could not be determined. *Acinetobacter baumannii* is a gram-negative coccobacillus commonly found in soil, water and dry environments [13], which has become a common and widespread bacterium in the hospital environment, due to its ability to persist for prolonged periods [13]. *Acinetobacter baumannii* is a clinically significant pathogen that can cause serious infections, including respiratory tract infections, bacteremia, and urinary tract infections, in addition to a multi-resistance to antibiotics [14].

## Conclusion

Even though this study cannot confirm whether the bacterial infection was nosocomial or otherwise, the presented case highlights the need to perform blood cultures on malaria positive patients, especially when fever persists after parasites have been cleared. Awareness of possible concomitant infection of *Acinetobacter baumannii* and *Plasmodium* spp. is important in clinical diagnostic. In children with malaria, undetected bacteria may contribute to morbidity due to untreated bacterial infections. the fact that bacteria was not treated. With the current rise in resistance among important bacterial pathogens, the necessity for blood (and other specimens) cultures for the enrichment of bacteria cannot be ignored. The ability for laboratories to perform bacterial cultures is, therefore, a key to avert the current trend in antibiotic resistance, especially in rural hospital settings. Antimicrobial test results should guide treatment decisions for patients in Ghana to reduce the inappropriate use of broad-spectrum antibiotics. The development of rapid and cost-effective alternatives for the identification of bacterial (co-) infections could be a solution for low-resource settings, where lack of financial means and expertise do not allow blood cultures to be performed.

## Data Availability

All the data information analyzed during this study are available at KCCR.

## Abbreviations

BSI: Bloodstream infections
BCS: Blantyre Coma Score
bpm: Beats per minute
E score: Eye-opening
M score: Motor
V score: Vocal
SSA: Sub-Saharan Africa
WHO: World Health Organization
KCCR: Kumasi Center for Collaborative Research in Tropical Medicine
